# Tackling social anxiety with targeted brain stimulation: investigating the effects of transcranial static magnetic field stimulation on self-focused attention

**DOI:** 10.3389/fnbeh.2024.1373564

**Published:** 2024-03-13

**Authors:** Nozomi Tomita, Hiroki Katayama, Yuto Kurihara, Toru Takahashi, Sumiya Shibata, Tatsuya Mima, Rieko Osu, Hiroaki Kumano

**Affiliations:** ^1^Educational Psychology, Tokyo Gakugei University, Koganei, Japan; ^2^Comprehensive Research Organization, Waseda University, Tokyo, Japan; ^3^Koto Child Guidance Office, Bureau of Social Welfare and Public Health, Tokyo Metropolitan Government, Koto, Japan; ^4^Faculty of Human Sciences, Waseda University, Tokorozawa, Japan; ^5^Laureate Institute for Brain Research, Tulsa, OK, United States; ^6^Japan Society for the Promotion of Science, Tokyo, Japan; ^7^Department of Physical Therapy, Niigata University of Health and Welfare, Niigata, Japan; ^8^The Graduate School of Core Ethics and Frontier Sciences, Ritsumeikan University, Kyoto, Japan

**Keywords:** social anxiety, attention, prefrontal cortex, magnetic field, near-infrared spectroscopy, speech

## Abstract

Previous studies suggested that self-focused attention (SFA), implicated in social anxiety disorder (SAD), correlates with heightened activity in the right frontopolar area (rFPA), which is the right prefrontal cortex just behind the forehead. Transcranial static magnetic field stimulation (tSMS) is a non-invasive brain stimulation method capable of temporarily suppressing brain function beneath the magnet. We explored whether tSMS on individuals with tendencies toward SAD elicited (1) suppressing rFPA activation during the resting-state and (2) reducing SFA during a subsequent speech task. Twenty-three university students with social anxiety performed two speech tasks. Between tasks, the tSMS group received neodymium magnet stimulation while the sham group received fake magnet stimulation on the rFPA for 20 min. Resting-state rFPA activities was measured using functional near-infrared spectroscopy (fNIRS), while SFA (body sensations and observer perspective), field perspective, and detached mindfulness (DM) perspective were assessed via questionnaires during both speech tasks. The observer perspective means SFA to self-imagery from others’ viewpoint, while the field and DM perspectives mean appropriately focusing on the external environment. The results indicated that tSMS intervention decreased rFPA activity from pre- to post-intervention rest. Then, tSMS reduced SFA to bodily sensations and increased DM perspective from pre- to post-intervention speech, especially in those with high levels of social anxiety. Furthermore, tSMS enhanced the field perspective regardless of social anxiety tendency. The results suggest that tSMS may suppress overactivity in rFPA, reduce SFA to body sensation, and increase adaptive attention in highly socially anxious individuals. Our study suggests the possibility of the clinical application of tSMS for treating SAD.

## 1 Introduction

Social anxiety disorder (SAD) is characterized by a marked fear of social situations in which others may scrutinize an individual (American Psychiatric Association, 2013). Cognitive-behavioral models of social anxiety propose that self-focused attention (SFA) is central to social fear (Clark and Wells, 1995), which involves focusing on inner cues such as negative thoughts, negative self-imagery, and bodily sensations (e.g., heart rate, blushing, and sweating).

Furthermore, researchers identify the *observer perspective* as a critical component of SFA, in which individuals see themselves as though from another person’s viewpoint (Clark and Wells, 1995). Although this mental image is typically negative, individuals tend to believe that it is the actual image seen by others because it is viewed from an observer’s perspective (McEwan and Devins, 1983). SFA lead to anticipated anxiety about a social situation, state anxiety, frequent negative thoughts, safety behaviors, and low self-evaluation of performance (Woody and Rodriguez, 2000; Bögels and Lamers, 2002; Spurr and Stopa, 2003; George and Stopa, 2008).

In order to reduce observer perspective, it is considered important to adopt a *field perspective*, in which their image of the situation is perceived as if they are viewing inside the scene with their own eyes, observing the details around them. Furthermore, metacognitive therapy (Wells and Matthews, 1994) emphasizes that acquiring a perspective called *detached mindfulness* (DM), in which one can observe various objects (including oneself) from a distant and objective viewpoint, is crucial for reducing SFA.

Regarding the neuroimaging research about SFA, Boehme et al. (2015) showed that SFA is linked to fMRI activation in the medial prefrontal cortex (MPFC), temporoparietal junction, and temporal pole by manipulating SFA during a simulated social situation. Moreover, Pujol et al. (2013) found that individuals with SAD patients exhibited greater activation in the primary visual cortex, deactivation in the dorsal frontoparietal, and small activation in anterior cingulate cortices when watching videos about themselves compared to unknown persons. However, those studies instructed participants to watch videos passively in social situations without real-time conversations.

By using functional near-infrared spectroscopy (fNIRS), which is less restrictive than fMRI, Tomita et al. (2020) and Tomita and Kumano (2021) examined brain regions associated with SFA in real-time social scenarios. Tomita et al. (2020) found increased activity in the right frontopolar area (rFPA), which is the right prefrontal cortex just behind the forehead and covers the MPFC, and in the right dorsolateral prefrontal cortex (rDLPFC) during the SFA condition (performed speech under the SFA instruction) compared to the control condition. Furthermore, Tomita and Kumano (2021) investigated whether rFPA and rDLPFC activity increased in individuals with high levels of social anxiety tendencies without SFA manipulation. The participants performed speech tasks under the no-instruction and control (looking at various places) conditions. The results showed that the higher the participants’ social anxiety, the more rFPA activity they showed in the no-instruction condition compared to the control condition. Additionally, increased rFPA activity from the control condition to the no-instruction was associated with higher participants’ social anxiety and higher subjective SFA.

In their third study, Tomita et al. (2021) conducted a speech task targeting individuals with social anxiety tendencies, using the same social settings as those of the previous studies (Tomita et al., 2020; Tomita and Kumano, 2021) before and after two-week exercise of the attention training technique and measured brain activity during the speech task. The results also showed that rFPA was activated in the no-instruction condition relative to the control condition, and rFPA activity from the control condition to the no-instruction was positively associated with subjective SFA.

These results (Tomita et al., 2020, 2021; Tomita and Kumano, 2021) suggested that when people speak publicly in social settings, greater oxy-Hb responses in the rFPA could be used as an objective marker of SFA in people with higher social anxiety. The term “marker” implies a one-to-one psychophysiological relationship between brain activity in the rFPA and SFA under specific circumstances (Cacioppo et al., 2017). Nonetheless, whether the linkage between these two variables is dependent or interdependent remains to be determined. The former scenario suggests that changes in the rFPA merely result from SFA, while the latter suggests a reciprocal relationship where rFPA activity could also contribute to the change of SFA. If the interdependent model holds, mitigating rFPA activity during public speaking in social settings could reduce SFA, a hypothesis that has yet to be explored.

Alongside neuroimaging research, the development of non-invasive brain stimulation (NIBS) techniques has substantially enriched our understanding of human brain function across the last decades (Tanaka et al., 2023). NIBS techniques such as transcranial magnetic stimulation (TMS) and transcranial current stimulation (tCS) have gained attention in neuropsychiatry as they offer a safe and non-surgical approach to manipulate neural circuits involved in neuropsychiatric disorders (Battaglia et al., 2023). Unlike neuroimaging, which offers correlational insights into the structure–function relationships within the brain, NIBS techniques provide evidence of the causal importance of specific brain regions in relation to targeted functions (Tanaka et al., 2023).

Transcranial static magnetic field stimulation (tSMS) using a small and strong neodymium (NdFeB) magnet can temporarily suppress brain function below it. It is a promising NIBS method due to its competitive advantages, such as safety, simplicity, and low cost (Oliviero et al., 2011; Nojima et al., 2020). It is reported that tSMS can suppress the cortical excitability in the human primary motor cortex (Oliviero et al., 2011), somatosensory (Kirimoto et al., 2016), and visual areas (Gonzalez-Rosa et al., 2015). These reports suggest the potential for a broad range of brain functional modulation effects. Therefore, if tSMS effectively suppresses the hyperactivity of the rFPA, the translational research of tSMS would extend to association cortices and related psychological functions. In addition, if tSMS could then attenuate SFA, this study would fill existing gaps in the current understanding of the linkage between the brain activities of the rFPA and SFA in social settings, explicitly showing the nature of their relationship, whether reciprocal or otherwise.

Katayama et al. (2021) preliminary investigated the possibility that suppressing the overactivation of the rFPA with tSMS would lead to the mitigation of SFA. Although previous studies have confirmed the safety of tSMS, this was the first research implementing tSMS on the rFPA. Therefore, the purpose was to confirm its safety for application to this brain area and to understand whether effectiveness could be observed through changes in brain activities, psychological indices, and qualitative information such as interviews with each participant. The study involved a small number of participants not screened for social anxiety and divided into two groups: tSMS (*n* = 3) and sham stimulation (*n* = 2). The participants engaged in speech tasks before and after stimulation. Changes in their cerebral activities with fNIRS and psychological indices during speech tasks were examined; however, statistical tests could not be conducted due to the small sample size. The participants were instructed to engage in SFA before each speech task to examine whether tSMS could suppress induced SFA.

By individually assessing changes in each indicator before and after stimulation, rFPA activity in the tSMS group decreased in five of the six channels covering rFPA. In contrast, the sham group displayed a mixed pattern of increase and decrease. Then, the study observed moderate decreases in SFA in the only tSMS group. Levels of state anxiety decreased for all participants, but the reduction was more pronounced for the tSMS group. These results suggested that tSMS may decrease the activation of the rFPA and mitigate SFA with related psychological indices. However, owing to the small sample size, the inability to conduct statistical tests, and the lack of screening for social anxiety, there was a substantial risk of drawing arbitrary conclusions from the limited data.

These preliminary results suggest several avenues for improvement for future investigation apart from the apparent necessity of increasing the number of participants. First, given that instructional manipulation may involve not only SFA but also an effort to control attention in general, cognitive processes such as intentional attention trying to follow instructions other than SFA may contaminate intervention effects. A critical advancement would be demonstrating that tSMS can diminish SFA in individuals with social anxiety without explicit directives, paving the way for clinical application. Second, NIRS is limited to measuring relative, rather than absolute, changes in brain activity. Our methodology involved a block design, alternating between rest and speech periods, using the preceding rest period as a baseline for calculating changes in oxy-Hb concentration during speech. However, considering the persistent inhibitory effect on brain activity for at least 30 min following a 30 min tSMS session (Dileone et al., 2018), tSMS could decrease brain activity not just during speech periods but also during baseline rest periods. This potential variation in brain activity across both speech and rest phases complicates the accurate assessment of tSMS’s effects on task-related brain activity changes when measured against baseline levels.

Additionally, resting-state brain activity may offer a clearer insight into the effects of tSMS on rFPA activity than measurement taken during speech periods. The speech tasks used the same topic for all the participants, and a certain time was given to contemplate the content of the speech; anyone could deliver a speech without difficulty. However, there may be a certain degree of variability in participants’ speech content and public speaking skills, significantly increasing the variability in rFPA activity taken during speech periods compared to rest periods. Although NIRS does not provide absolute values, assessing the relative change in resting-state brain activities from before to after an intervention can yield valuable information. Consequently, in this study, we focused on evaluating changes in the resting-state cerebral activities preceding each speech period during the speech tasks.

This study aims to investigate the effects of tSMS on individuals with tendencies toward social anxiety and address two specific objectives. First, we investigate whether tSMS suppresses rPFA activities during the resting state compared with sham stimulation. Second, we investigate whether tSMS improves SFA scores (body sensations and self-imagery from the observer perspective) and the relevant psychological indices during the speech task compared with sham stimulation. NIBS in preclinical research allows scientists to explore the neural circuitry involved in neuropsychiatric conditions, providing valuable insights into the underlying pathology and facilitating the assessment of the safety and efficacy of these techniques before translating them into clinical applications (Tanaka et al., 2023). This preclinical research contributes a clearer understanding of the relationship between SFA in social settings and rFPA activity. It assesses the safety and efficacy of tSMS for social anxiety before translating them into clinical applications.

## 2 Method

### 2.1 Participants

We recruited 23 young adults with social anxiety tendencies (women: 12, men: 11), aged 20.81 ± 1.62 years, by handing out an application form to university students in 2022. Before the experiment, the participants answered the Japanese version of the Liebowitz Social Anxiety Scale (LSAS-J: Liebowitz, 1987; Japanese version: Asakura et al., 2002) with Google Forms. Using receiver operating characteristics (ROC) analyses, it was found that a score of 30 on the self-report version of the LSAS provided the best balance of sensitivity and specificity for differentiating patients with SAD from healthy controls. Similarly, a score of 60 provided the best balance of sensitivity and specificity for classifying patients with generalized and nongeneralized SAD according to DSM-IV (Mennin et al., 2002). We screened the participants for a boundary score of 30, which pertains to the score mentioned above and usually refers to the not symptom-free level of the LSAS-J. In addition, the participants again answered the LSAS-J on the day of the experiment, and we reconfirmed that each participants’ LSAS-J scores was higher than 30. The participants were Japanese, right-handed, and reported no psychological disorders, hearing problems, or neurological or cardiovascular illnesses. Furthermore, no participants reported poor physical condition, lack of sleep, any medication within 24 h, or alcohol and caffeine consumption within 12 h prior to the beginning of the experiment. We randomly allocated the participants into the tSMS and sham groups.

### 2.2 Self-report measures

#### 2.2.1 Social anxiety

The Japanese version of the Liebowitz Social Anxiety Scale (LSAS-J) measures the severity of social anxiety and assesses fear and avoidance of 24 common situations related to social performance and interaction (Liebowitz, 1987; Japanese version: Asakura et al., 2002). The participants rated the extent to which they agreed with each statement using a four-point Likert-type scale from 0 = not at all to 3 = totally. The scores range from 0 to 144. Previously, the cut-off point on the LSAS-J was determined to be 44 (Asakura et al., 2002). The LSAS-J has high internal consistency (*α* = 0.95) and test–retest reliability (0.92). The LSAS-J scores of patients with SAD were correlated with the Japanese version of the Social Avoidance and Distress Sale (*r* = 0.65, *p* < 0.001) and severity scores assessed by a medical doctor (*r* = 0.74, *p* < 0.001; Ishikawa et al., 1992). Thus, the reliability of the LSAS-J and its validity for measuring clinically significant anxiety have been demonstrated (Asakura et al., 2002).

#### 2.2.2 Self-focused attention to body sensations

The Focused Attention Scale (FAS) was developed based on the Focused Attention Questionnaire (Chambless and Glass, 1984) and comprised three items translated from the questionnaire and nine original items (Yamada et al., 2002). The FAS has two subscales: FAS-self (six items) measures SFA to body sensations, and FAS-others (six items) assesses other-focused attention (OFA). OFA, directed at environmental threats such as evaluation by others, is distinct from SFA and is another maintaining factor in SAD. We only used the FAS-self subscale to measure SFA to body sensations. The participants rated the degree to which they agreed with each statement using a five-point Likert-type scale (1 = not at all; 5 = totally). The range of each subscale is 1 to 30. The FAS-self demonstrated adequate internal consistency (*α* = 0.76), and FAS-self has been correlated with the Social Avoidance and Distress Scale (*r* = 0.36, *p* < 0.01) and the Fear of Negative Evaluation Scale (Ishikawa et al., 1992; *r* = 0.32, *p* < 0.01). Thus, the validity and reliability of the FAS have been demonstrated (Yamada et al., 2002).

#### 2.2.3 Observer, field, and detached mindfulness perspectives

The Mental Perspective Scale for Social Anxiety Disorder (MPS) comprises three subscales, namely, field (MPS-F; five items, the range is 1 to 30), observer (MPS-O; four items, the range is 1 to 24), and detached mindfulness (MPS-DM; four items, the range is 1 to 24) perspectives (Tomita et al., 2018). The participants rated their level of agreement with each statement using a six-point Likert-type scale ranging from 1 = not at all to 6 = totally. High scores in MPS-F and MPS-DM indicate a more adaptive mental perspective, whereas high scores in MPS-O suggest a propensity for maladaptive mental perspective. MPS-O measures SFA to self-image from the observer perspective, while MPS-F and MPS-DM measure adaptive attention that counters the observer perspective. Each subscale has exhibited adequate internal consistency (MPS-F: *α* = 0.81; MPS-O: *α* = 0.73; MPS-DM: *α* = 0.77) and test–retest reliability (MPS-F: 0.73; MPS-O: 0.63; MPS-DM: 0.58). The study found that MPS-F is correlated with *divided attention* (Imai et al., 2015; *r* = 0.33, *p* < 0.05), while MPS-O is correlated with the Fear of Negative Evaluation Scale (*r* = 0.64, *p* < 0.01). Lastly, MPS-DM is correlated with *attention switching* (*r* = 0.32, *p* < 0.05), *divided attention* (*r* = 0.35, *p* < 0.05), and the Detached Mindfulness Mode Scale (Imai and Kumano, 2021; *r* = 0.28, *p* < 0.05). Thus, the validity and reliability of the MPS have been demonstrated (Tomita et al., 2018).

#### 2.2.4 State anxiety

The participants rated the degree of state anxiety at the beginning of the experiment and during the speech tasks with Visual Analog Scale (VAS) (from 0 = not at all to 100 = completely).

#### 2.2.5 Sleepiness

We used the Stanford Sleepiness Scale (SSS) to measure sleepiness at the beginning of the experiment (Hoddes et al., 1972).

#### 2.2.6 Handedness

We used the Flinders Handedness Survey to confirm whether the participants were right-handed (Nicholls et al., 2013; Japanese version: Okubo et al., 2014).

### 2.3 Functional near-infrared spectroscopy

fNIRS is a non-invasive method for monitoring brain hemodynamics, which enables studying brain function and various pathologies (Ferrari and Quaresima, 2012; Scholkmann et al., 2014). In contrast to fMRI and electroencephalography (EEG), which are sensitive to motion artifacts, fNIRS is less affected by movement and can measure brain function during activities such as speech tasks.

**Figure 1:**
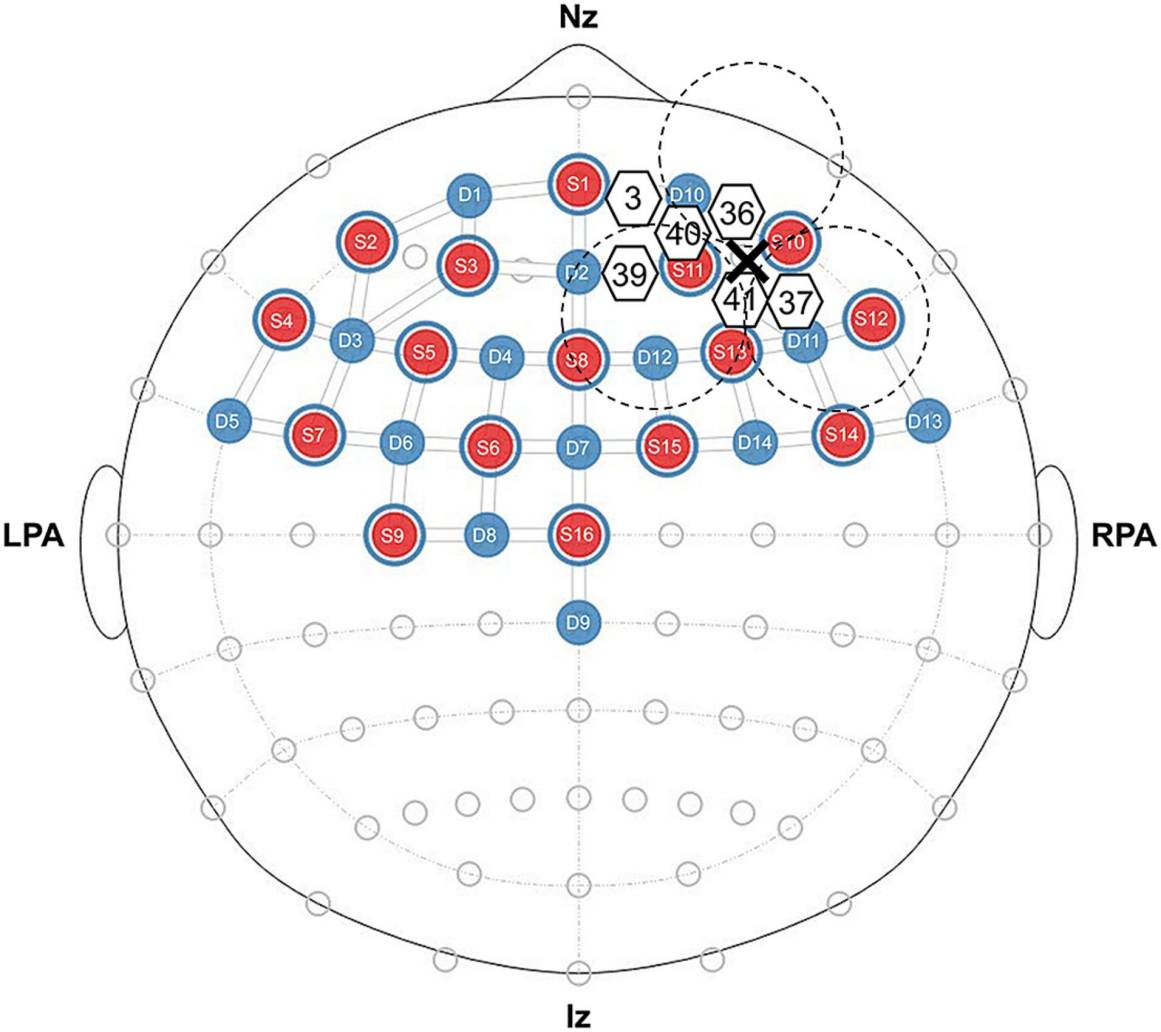
Near-infrared spectroscopy probe layout, regions of interest, and configuration diagram for real or sham tSMS. We present the source in red and the detector in blue, and we denote ROI channels using white-bordered numbers. The positioning of real and sham tSMS is depicted using dashed lines with a cross representing the center of the magnet.

We used an optical topography system (NIRSport2, NIRx Medical Technology, Germany) to measure brain activity during the resting state and speech task. We measured changes in cerebral oxy-Hb and deoxyhemoglobin (deoxy-Hb) concentrations at two wavelengths of near-infrared light (760 and 850 nm). The fNIRS setup used 16 LED light sources and 14 avalanche photodiode detectors with an inter-optode distance of approximately 30 mm, which forms 32 actual measurement channels (Figure 1). The measurement principles were based on the modified Beer–Lambert law (Baker et al., 2014), which calculates changes in oxy-Hb and deoxy-Hb concentrations from the change in light attenuation at a given measurement point. The study assumed a differential path length factor, and changes in oxy-Hb and deoxy-Hb concentrations were expressed in mmol × mm. The fNIRS cap was established by centering around the Cz position following the international 10–20 electrode system. According to the results of Tomita and Kumano (2021), the regions of interest (ROI) included the right PFC (CH3, CH36, CH37, CH39, CH40, and CH41) as depicted in Figure 1.

The participants were requested to refrain from making body movements apart from speaking during the speech tasks. Both pre-intervention and post-intervention speech used a block design consisting of three sets of a 60 s rest period in which the participants gazed at a fixation cross in the center of the screen and a 60 s speech period, followed by a 120 s final rest period.

We defined resting-state brain activity by averaging the accumulated data during the last 20 s of the three rest periods immediately before the subsequent speech periods in each pre-intervention and post-intervention speech. As outlined in the introduction, our decision to focus on resting brain activity rather than on activity during the speech period was driven by two essential considerations. First, due to the inherent limitations of NIRS in measuring absolute brain activity values, assessments during a speech period must rely on observing changes relative to a resting baseline. However, brain activity may be reduced not only during speech periods but also during the intended resting baseline periods. This potential modification of the resting baseline complicates accurate assessments of how stimuli influence changes in brain activity related to speech. Second, resting-state brain activity may reflect the direct effects of tSMS more accurately on rFPA activity, compared to measurements taken during speech periods, which additional speech-related activities may confound. The evaluation of relative changes in resting-state brain activity before and after an intervention is therefore deemed an effective method to assess the direct effects of tSMS on rFPA activity. Supporting this approach, a study by Deiters et al. (2013) found that individuals with high speech anxiety exhibited SFA not only during speeches but also in anticipation of speaking. That suggests that rFPA activity during rest periods is significantly related to SFA during speech periods, allowing for an assessment of whether tSMS can mitigate SFA during the anticipation of speeches.

### 2.4 Speech tasks

Under the same protocols as Tomita et al. (2020, 514), “the participants performed speech tasks in front of a monitor that displayed four audience members: two acted out positive and negative gestures, respectively, while the other two acted out neutral gestures.” The topic of their speech was their school life from elementary school to the present. Before each speech task, participants were given 5 min to contemplate the content of their speech. Although the participants were informed that the audience members displayed on the monitor were in the next room evaluating their speech in real-time, the audiences were prerecorded (Tomita et al., 2020). We used six videos in each speech to prevent the participants from recognizing that the audience members were not in another room while they delivered speeches. Each audience member was provided a clipboard for rating the speeches and acted out three gestures per minute, which were selected by referring to Perowne and Mansell (2002). Detailed information on the videos (e.g., the contents of the gestures of each audience member) is available from Tomita et al. (2020).

### 2.5 tSMS or sham intervention

We used a triple tSMS system with three cylindrical nickel-plated (Ni–Cu–Ni) NdFeB magnets placed close to one another (Shibata et al., 2022):

The north pole of the three magnets were embedded in a foundation made of non-magnetic material with a diameter of 140 mm and a thickness of 48 mm. The vertical axis of the magnets was tilted 16.5 degree from that of the foundation. Parameters of the magnets were as follows: the diameter was 50 mm, the thickness was 30 mm, the maximum energy density was 406 kJ/m^3^, the nominal strength was 863 N, and the surface magnetic flux density was approximately 5340 G.

The study used three magnets to resolve a trade-off between the summation of the magnetic fields from multiple magnets and the avoidance of poor focality. The triple tSMS system can produce an effective magnetic field in deep areas and modulate brain functions (Shibata et al., 2022). The current tSMS system with a single magnet system was insufficient to effectively stimulate deep brain areas due to the attenuation of the magnetic field proportionally to the distance from the magnet. In contrast, the triple tSMS system produced a magnetic field sufficient for neuromodulation up to 80 mm depth from the magnet surface, 30 mm deeper than the conventional tSMS system (Shibata et al., 2022). The triple tSMS is as safe as the conventional tSMS with a single magnet system. Because we aimed to target the rFPA, including the MPFC, we utilized the triple tSMS system.

The sham stimulation device was the same size, appearance, and sensation as the triple tSMS system, except three non-magnetic stainless-steel cylinders were embedded in the foundation. The stimulation was assigned randomly among the subjects, and they were blinded to the type of stimulation. Triple tSMS (or sham) was held using an arm-type light stand (Avenger C-stand and Super Clamp; Manfrotto, Cassola, Italy) over the representational field of the right frontpolar area (CH36, CH37, CH40, and CH41) as depicted in Figure 1. The intervention duration was set to 20 min.

### 2.6 Procedure

**Figure 2:**
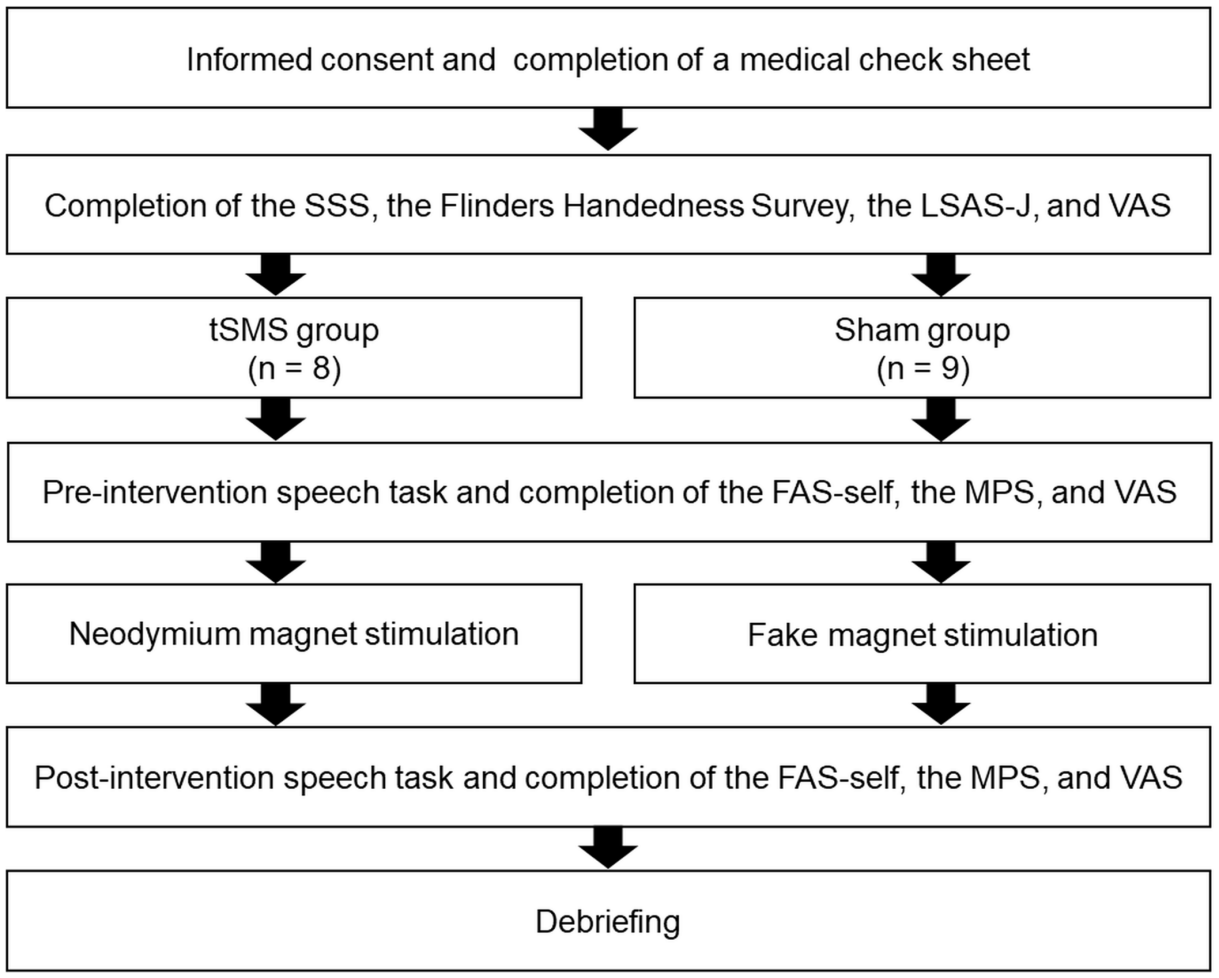
Experimental procedure. SSS, Stanford sleepiness scale; LSAS-J, the Japanese version of the Liebowitz social anxiety scale; VAS, visual analog scale of state anxiety; FAS-self, a subscale of the focused attention scale; MPS, mental perspective scale for social anxiety disorder.

We illustrated the procedure in Figure 2. The study was explained to the participants before signing a consent statement. Subsequently, they completed a medical check sheet, the SSS, and the Flinders Handedness Survey, of which we checked every answer. They then completed the LSAS-J and VAS to measure at the time of the experiment and baseline state anxiety, respectively. Next, the participants sat in front of a computer (PC: HP Probook 430 G5; display monitor: BenQ-GW2470-T, 24 in), in which the experimenter explained the speech tasks. The participants then prepared the content of their speech for 5 min. The participants were attached to the fNIRS probe holder, followed by conducting the pre-intervention speech task. Afterward, they evaluated their subjective degrees of SFA (body sensations and self-imagery from the observer perspective), adapted mental perspective, and state anxiety during the speech using FAS-self, MPS-O, MPS-F, MPS-DM, and VAS, respectively.

The fNIRS was removed temporarily, and neodymium magnets were applied to the headcaps of the tSMS group, whereas the headcaps of the sham group were attached with fake magnets for 20 min. We asked them to rest during the tSMS or sham stimulation, and verbal checking was done every 5 min to prevent them from falling asleep.

After intervention with the tSMS or sham stimulation, the participants conducted the second speech task while wearing the fNIRS. They then evaluated their subjective degrees of SFA (body sensations and self-imagery from the observer perspective), adaptive mental perspectives, and state anxiety during the speech using FAS-self, MPS-O, MPS-F, MPS-DM, and VAS, respectively. Finally, they were debriefed and asked whether they suspected the audience was not in the next room. After the experiment, we transferred the reward money (1,500 yen) into the designated accounts for participating in the study.

### 2.7 Hypotheses

The study posed four hypotheses. First, tSMS would decrease rFPA activities during the resting periods of the post-intervention speech more than those of the pre-intervention speech. Furthermore, since the higher the tendency for social anxiety, the greater the level of rFPA activity before intervention, the decrease in rFPA activity through tSMS would be more pronounced. Second, tSMS would decrease SFA scores, including SFA to body sensations and observer perspective, more during the post-intervention speech than during the pre-intervention speech. Furthermore, since the higher the tendency for social anxiety, the higher the score of SFA before intervention, these effects would be more pronounced in individuals with high levels of social anxiety. Third, tSMS would increase the scores of the field and detached mindfulness perspectives during the post-intervention speech more than during the pre-intervention speech. Furthermore, since the higher the tendency for social anxiety, the lower the score of the field and detached mindfulness perspectives before intervention, these effects would be more pronounced in individuals with high levels of social anxiety. Fourth, regarding changes between the pre-intervention speech and the post-intervention speech in the tSMS group, positive relationships will occur between the change in resting-state rFPA and the change in SFA scores, as well as negative relationships between the change in resting-state rFPA and the change in the scores of the field and detached mindfulness perspectives. Although this study investigated whether tSMS differently changes state anxiety during the two speech tasks, no hypothesis is formulated because tSMS may influence in the case that it reduces SFA.

### 2.8 Data preparation and analysis

#### 2.8.1 Exclusion criteria for data analysis

The criteria for excluding a participant’s data from the analysis were as follows: (1) after reassessment using the Flinders Handedness Survey, the participant was left-hand dominant. (2) The participant could not finish the speech task because the video was unexpectedly interrupted halfway through. (3) The participant was identified as an outliner using the Smirnov–Grubbs test (Grubbs, 1950; 1969). Lastly, (4) the participant’s LSAS-J score on the day of the experiment was lower than 30.

#### 2.8.2 Data preparation of near-infrared spectroscopy

Similar to previous studies (Tomita et al., 2020; Tomita and Kumano, 2021), we analyzed data only for oxy-Hb and not deoxy-Hb. We conducted data preparation using MATLAB [R2020b Update 4 (9.9.0.1570001)] on the Open Platform of Transparent Analysis Tools for fNIRS (Open PoTATo, Yamata et al., 2012). The study applied the filtering function of Open PoTATo, including opSSR (noise reduction through short-distance correction), motion check (omission of effects due to body movement), and band filter (application of a 0.1 Hz low-pass filter to eliminate noise generated by physiological activities such as heartbeats, respiration, and eye movement, and a 0.01 Hz high-pass filter to eliminate noise generated by physiological processes such as body temperature). The range of band filters was the same as Tomita et al. (2018), Tomita and Kumano (2021), and Wang et al. (2022). Meaningful variations in brain activity observed during the 20 s rest periods, measured in pre- and post-intervention speech tasks, are expected to fit within the scope of these filters.

#### 2.8.3 Statistical analysis

We used HAD17_105 (Shimizu, 2016) for analyses. As a manipulation check, we submitted the VAS of state anxiety to a 2 (group: tSMS versus sham) × 2 (time: at the start of the experiment versus during the pre-intervention speech) mixed-design analysis of variance with repeated measures on the second variable.

To investigate the first hypothesis, we performed multiple regression analyses based on the generalized linear model (GLM). The predictor variables were group (tSMS and sham), LSAS-J score, and the interaction between group and LSAS-J; the outcome variable was the Δoxy-Hb for each channel within ROI (resting-state brain activity in the post-intervention speech minus that in the pre-intervention speech). The result indicates that as the outcome values (Δoxy-Hb) become increasingly negative, a larger decrease occurs in brain activity after the intervention with tSMS or sham stimulation than before the intervention. Regarding the group, the sham was converted to 0, while the tSMS was converted to 1 as a dummy variable. When the interaction between the group and LSAS-J was shown in each analysis, we used simple slope analysis using the data of ±1SD of LSAS-J to plot the interaction pattern.

To investigate the second and third hypotheses, we performed multiple regression analyses based on GLM. The predictor variables were group (tSMS and sham), LSAS-J score, and the interaction between the group and LSAS-J. The outcome variable was ΔFAS-self, ΔMPS, and ΔVAS (the scores obtained in the post-intervention speech minus that in the pre-intervention speech). This finding indicates that as the outcome values (Δ) become increasingly negative, the scores decreased compared with before the intervention. In contrast, it indicates that as the outcome values (Δ) become increasingly positive, the scores increased compared with before the intervention. Regarding the group, the sham was converted to 0, while the tSMS was converted to 1 as a dummy variable. When the interaction between LSAS-J and the group was shown, we used simple slope analysis using the data of ±1SD of LSAS-J to plot the interaction pattern.

To investigate the fourth hypothesis, we calculated Spearman’s rank correlation coefficient between Δoxy-Hb and Δpsychological questionnaires, which were significantly affected by the tSMS.

### 2.9 Compliance with ethical standards

#### 2.9.1 Ethical approval

All procedures performed in studies involving human participants were in accordance with the ethical standards of the institutional and national research committee (Ethics Review Committee on Research with Human Subjects, 2019-283) and with the 1964 Helsinki Declaration and its later amendments or comparable ethical standards.

#### 2.9.2 Animal rights

This article does not contain any studies with animals performed by any of the authors.

#### 2.9.3 Informed consent

Informed consent was obtained from all participants. The datasets generated during and analyzed in the current study are available from the corresponding author upon reasonable request.

## 3 Results

### 3.1 Participant characteristics

One, three, and two participants were excluded by the first, second, and third criteria, respectively. The fourth criterion excluded none. We used the data on the remaining 17 participants for analyses. The tSMS and sham groups comprised 8 and 9 participants, respectively.

### 3.2 Experimental manipulation check

We submitted the VAS of state anxiety to a 2 (group: tSMS versus sham) × 2 (time: at the start of the experiment versus during the pre-intervention speech) mixed-design ANOVA with repeated measures on the second variable. The result indicates that the main effect of time was significant (*F* (1, 15) = 30.711, *p* = 0.0001). The VAS of state anxiety during the pre-intervention speech was higher than that at the start of the experiment. Therefore, the study confirmed that the speech task induced anxiety appropriately.

### 3.3 Hypothesis 1: changes in brain activities

**Figure 3:**
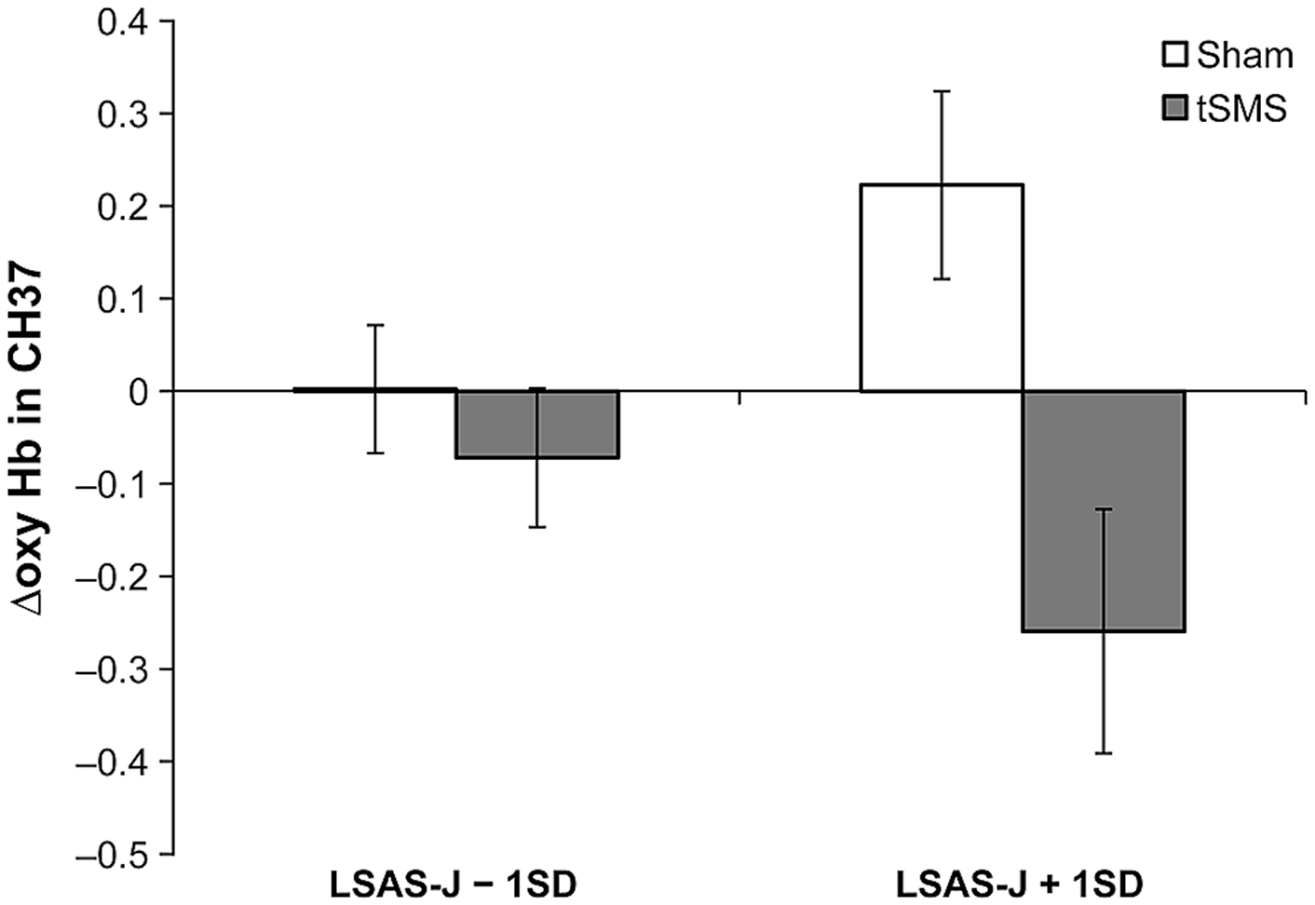
Results of simple slope analysis using the data of ±1 SD of the LSAS-J to plot the interaction pattern of Δoxy Hb in CH37. This figure indicates the means and standard errors of Δoxy-Hb in CH37 for each condition when adjusting the LSAS-J scores for all participants to LSAS-J + 1 SD or LSAS-J − 1 SD values. The results indicate that as the outcome values (Δoxy-Hb) become increasingly negative, a large decrease in brain activity occurs, following intervention with the tSMS or sham stimulation compared with that before the intervention. LSAS-J, the Japanese version of the Liebowitz social anxiety scale.

Table 1 presents the results of the multiple regression analyses based on GLM. The main effects of the group significantly predicted Δoxy-Hb in CH36, CH37, CH39, and CH41. ΔOxy-Hb in CH36, CH37, CH39, and CH41 in the tSMS group were lower than in the sham group. Furthermore, the interaction between the group (tSMS versus sham) and LSAS-J significantly predicted Δoxy-Hb in CH37. When LSAS-J was at +1SD, Δoxy-Hb in the CH37 was significantly lower in the tSMS group than in the sham group (Figure 3; LSAS-J + 1SD: *b* = −0.482, *β* = −0.846, SE = 0.152, *p* = 0.001; LSAS-J − 1SD: *b* = −0.074, *β* = −0.130, SE = 0.123, *p* = 0.547). Regarding Δoxy-Hb in CH3 and CH40, the main effects of the group and LSAS-J and their interaction were nonsignificant.

**Table 1:** Results of the multiple regression analysis of changes in brain activities.

Outcome	Predictor	*β*	*p*
ΔCH3	Group (tSMS versus sham)	−0.227	0.304
	LSAS-J	−0.240	0.197
	Interaction	0.049	0.781
	*R*^2^	0.152	0.310
ΔCH36	Group (tSMS versus sham)	−0.415	0.035^*^
	LSAS-J	−0.072	0.635
	Interaction	−0.230	0.107
	*R*^2^	0.252	0.093^†^
ΔCH37	Group (tSMS, sham)	−0.488	0.007^**^
	LSAS-J	0.028	0.857
	Interaction	−0.326	0.026^*^
	*R*^2^	0.345	<0.001^***^
ΔCH39	Group (tSMS, sham)	−0.505	0.006^**^
	LSAS-J	−0.154	0.199
	Interaction	0.028	0.807
	*R*^2^	0.113	0.032^*^
ΔCH40	Group (tSMS, sham)	−0.313	0.095^†^
	LSAS-J	−0.209	0.282
	Interaction	0.096	0.597
	*R*^2^	0.201	0.138
ΔCH41	Group (tSMS, sham)	−0.515	0.008^**^
	LSAS-J	−0.113	0.561
	Interaction	−0.184	0.312
	*R*^2^	0.352	0.040^*^

LSAS-J, the Japanese version of the Liebowitz social anxiety scale; *β*, standardized partial regression coefficient. ^†^*p* < 0.10, *p < 0.05, **p < 0.01, and ***p < 0.001.

### 3.4 Hypotheses 2: changes in self-focused attention

**Figure 4:**
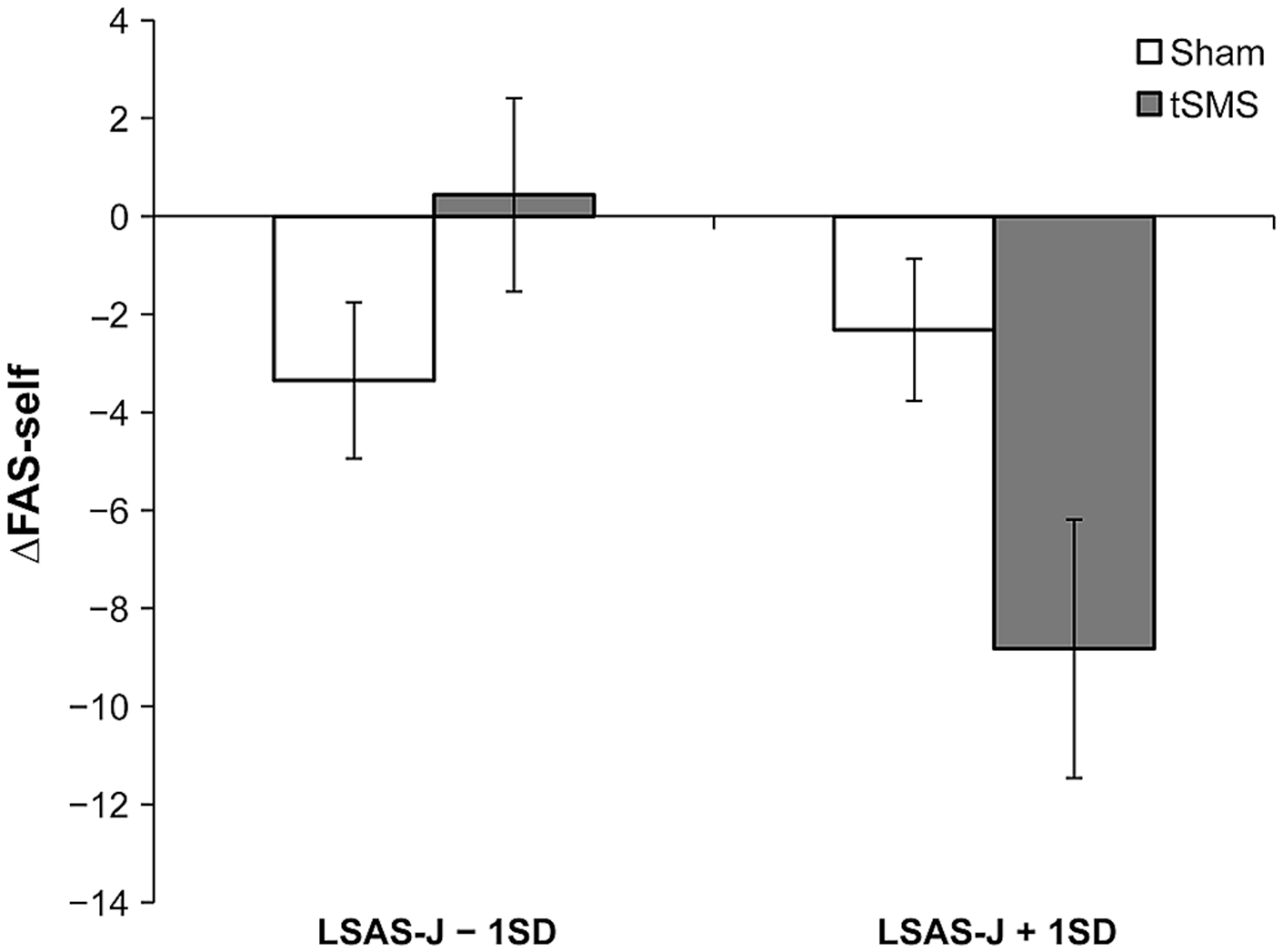
Results of simple slope analysis using the data of ±1SD of LSAS-J to plot the pattern of interaction of ΔFAS-self. This figure indicates the means and standard errors of ΔFAS-self for each condition when adjusting the LSAS-J scores for all participants to LSAS-J + 1 SD or LSAS-J − 1 SD values. The results indicate that as the outcome values become increasingly negative, a decrease occurs in self-focused attention relative to before the intervention. LSAS-J, the Japanese version of the Liebowitz social anxiety scale; FAS-self, the subscale of focused attention scale.

Table 2 provides the results of multiple regression analyses based on GLM for subjective measurements. Although the main effect of the group was not significant, the results demonstrated that the interaction between group and LSAS-J significantly predicted ΔFAS-self. When LSAS-J was at +1SD, ΔFAS-self was significantly lower for the tSMS than for the sham group (Figure 4; LSAS-J + 1SD: *b* = −6.507, *β* = −0.570, SE = 3.294, *p* = 0.048; LSAS-J − 1SD: *b* = 3.786, *β* = 0.332, SE = 2.152, *p* = 0.079). The main effect of the LSAS-J significantly predicted ΔFAS-self, which was lower when the LSAS-J was lower. Regarding ΔMPS-O, the main effects of the group and LSAS-J and their interaction were nonsignificant.

**Table 2:** Results of multilevel multiple regression analysis on changes in subjective measurements.

Outcome	Predictor	*β*	*p*
ΔFAS-self	Group (tSMS versus sham)	−0.119	0.517
	LSAS-J	−0.350	0.024^*^
	Interaction	−0.410	0.005^**^
	*R*^2^	0.303	0.015^*^
ΔMPS-F	Group (tSMS versus sham)	0.420	0.018^*^
	LSAS-J	0.087	0.673
	Interaction	0.063	0.745
	*R*^2^	0.214	0.083^†^
ΔMPS-O	Group (tSMS versus sham)	−0.154	0.525
	LSAS-J	0.208	0.433
	Interaction	−0.146	0.556
	*R*^2^	0.074	0.334
ΔMPS-DM	Group (tSMS versus sham)	0.616	0.001^**^
	LSAS-J	0.172	0.240
	Interaction	0.296	0.031^*^
	*R*^2^	0.568	<0.001^***^
ΔVAS	Group (tSMS versus sham)	−0.452	0.074^†^
	LSAS-J	0.031	0.893
	Interaction	−0.344	0.115
	*R*^2^	0.322	0.002^**^

LSAS-J, the Japanese version of the Liebowitz social anxiety scale; FAS-self, the subscale of focused attention scale; MPS-F, field perspective of mental perspective scale for social anxiety disorder; MPS-O, observer perspective of MPS; MPS-DM, detached mindfulness perspective of MPS; VAS, visual analog scale of state anxiety; *β*, standardized partial regression coefficient; ^†^*p* < 0.10, *p < 0.05, **p < 0.01, and ***p < 0.001.

### 3.5 Hypothesis 3: changes in the relevant psychological indices

**Figure 5:**
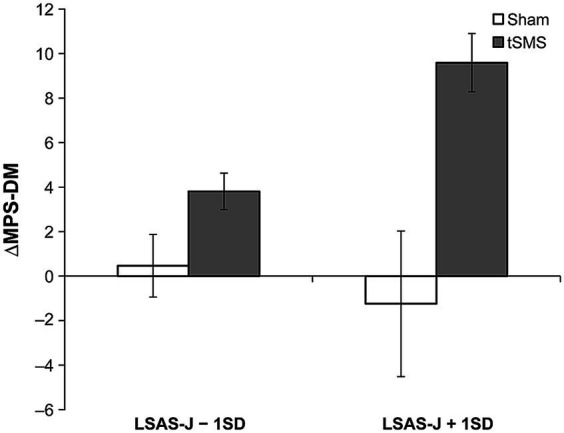
Results of simple slope analysis using the data of ±1 SD of the LSAS-J to plot the pattern of interaction of ΔMPS-DM. This figure indicates the means and standard errors of ΔMPS-DM for each condition when adjusting the LSAS-J scores for all participants to LSAS-J + 1 SD or LSAS-J − 1 SD values. As the outcome values become increasingly positive, an increase occurs in the DM perspective compared with before the intervention. LSAS-J, the Japanese version of the Liebowitz social anxiety scale; MPS-DM, detached mindfulness perspective of mental perspective scale for social anxiety disorder.

The main effect of the group significantly predicted the ΔMPS-F and ΔMPS-DM, in which ΔMPS-F and ΔMPS-DM in the tSMS group were higher than those of the sham group. Furthermore, the results indicated that the interaction between the group and LSAS-J also significantly predicted ΔMPS-DM. When LSAS-J was at +1SD, ΔMPS-DM was significantly higher in the tSMS than in the sham group (Figure 5, LSAS-J + 1SD: *b* = 10.835, *β* = 0.942, SE = 1.540, *p* < 0.001; LSAS-J − 1SD: *b* = 3.339, *β* = 0.290, SE = 3.562, *p* = 0.348). Regarding ΔVAS, the main effects of the group and LSAS-J and their interaction were nonsignificant.

### 3.6 Hypothesis 4: relationship between changes in brain activities and subjective measurements

For CH36, CH37, CH39, CH41, FAS-self, MPS-F, and MPS-DM, in which significant main effects of the group or interactions between the group and LSAS-J were observed, the study calculated Spearman’s rank correlation coefficients only in the tSMS group to explore relationships between the changes in these measures. The results showed that only Δoxy-Hb in CH41 and CH36 were relatively strongly correlated with ΔFAS-self (*ρ* = 0.683, *p* = 0.06) and ΔMPS-DM (*ρ* = −671, *p* = 0.07), respectively. However, these relationships did not reach statistical significance due to the small sample size.

## 4 Discussion

This study aimed to investigate the effects of tSMS on the resting-state activity of the right frontopolar area (rFPA), self-focused attention (SFA), and other related psychological indices during a speech in individuals with social anxiety tendency. As a result, CH36, CH37, CH39, and CH41 displayed decreased resting-state brain activity with tSMS. Particularly regarding CH37, this effect was prominent in individuals with high levels of social anxiety. Regarding SFA and other psychological indices, tSMS reduced SFA to bodily sensations and improved the detached mindfulness (DM) perspective, especially in individuals with high levels of social anxiety tendency. Additionally, tSMS improved the field perspective regardless of social anxiety tendency. In the tSMS group, the study observed relatively large but nonsignificant positive and negative correlations between the change in SFA for body sensation and the change in resting-state brain activities in CH41 and between the change in DM perspective and the change in resting-state brain activities in CH36, respectively. Therefore, the results supported the first hypothesis for four out of six channels. The results further supported the second hypothesis only for SFA to body sensations. Meanwhile, the third was proved for the field and DM perspectives, and the fourth was affirmed for only two out of 12 potential relationships.

The first hypothesis regarding the CH3 and CH40 was not supported. CH3 might not receive sufficient magnetic force as the centers of the magnets were surrounded by CH36, CH37, CH40, and CH41, as depicted in Figure 1. Further, a previous study demonstrated that although the triple tSMS system could provide the same neuromodulatory effect as the conventional tSMS, the magnetic field just below the triple tSMS system was weaker than the conventional tSMS, due to the interference of the magnetic fields produced by the three magnets (Shibata et al., 2022). Since CH40 was positioned just at the boundary of two magnets, its activity may not have been suppressed due to interference of the magnetic fields, which will be a topic for future investigation.

Regarding the second and third hypotheses, individuals with high scores for LSAS-J exhibited a greater improvement in SFA to body sensations and the DM perspective. This observation can be contextualized through Gonzalez-Rosa et al. (2015), which found a significant impact of tSMS on the visual field during challenging tasks. Likewise, individuals with high LSAS-J scores may experience more SFA during speech tasks and more difficulty delivering a speech. Then, tSMS may be particularly effective in addressing these difficulties, which can be a promising result for future clinical applications to SAD.

Although the clinical effectiveness of tSMS for post-stroke hemiparesis has been proved by a randomized controlled study (Shimomura et al., 2023), no conclusive data supports its usefulness in psychiatric disorders. In this regard, the present study in higher socially anxious individuals is significant because it demonstrated the suppressive effects of tSMS on the activity of the rPFA during the resting state and its potential to inhibit SFA, which might be a relevant factor for the pathogenesis of SAD. Furthermore, it is conceptually novel that tSMS not only suppresses maladaptive cognitive processes but also enhances adaptive cognitive processes like the DM and field perspectives. Specifically, the DM perspective demonstrated a more pronounced change than FAS-self, as indicated by contribution rates and *p*-values in multiple regression analysis. Enhancing the DM perspective appears crucial when implementing tSMS for SAD.

In contrast, tSMS did not decrease the MPS-O score (SFA to self-imagery from the observer perspective) during speech tasks. The lack of a decrease in the observer perspective may be because rFPA is linked with SFA related to body sensations, but it is indirectly connected to the observer perspective. Tomita and Kumano (2021) found that the interaction between social anxiety and SFA to body sensation during the speech task was significantly associated with rFPA activity. However, the interaction between social anxiety and SFA of the observer perspective was not. As noted, when SAD patients watched a video about themselves with observer perspective, they displayed significantly greater activation in the primary visual cortex than controls and a significant deactivation or small activation in the dorsal frontoparietal and anterior cingulate cortices (Pujol et al., 2013). Therefore, it may be helpful to investigate the effect of tSMS on the primary visual cortex to examine whether it can suppress the observer perspective of individuals with SAD.

tSMS did not decrease the visual analog scale score about state anxiety during speech tasks. There would be no change in state anxiety because the brain region associated with state anxiety is mainly the insula, not the rPFA (Baur et al., 2013). In cognitive-behavioral therapy, it is deemed more crucial to target process variables that maintain SAD rather than solely focusing on lowering state anxiety. Therefore, the intriguing finding of the present study is that only the cognitive process of SFA corresponding to the rPFA changed with tSMS, despite the absence of a change in state anxiety.

To summarize, applying tSMS to the rFPA did not necessarily alleviate all features that constituted SFA, as evidenced by the significant reduction in SFA to body sensation but not in SFA of the observer perspective. On the other hand, not only were maladaptive features suppressed, but adaptive states such as the field and DM perspectives were also enhanced. While the field and observer perspectives have been considered to lie on the same axis with opposite directions, the lack any significant reduction in observer perspective suggests that they are not necessarily aligned on the same axis. Additionally, the DM perspective has been considered conceptually distinct from the observer perspective, implying a broader concept including mindfulness and attention control (Tomita et al., 2018), and the relationship between the rFPA and the DM perspective has never been investigated. Therefore, the fact that the DM perspective was more strongly associated with the rFPA than the observer perspective warrants further study.

Regarding the probable support for the fourth hypothesis, the results suggest that the suppression of brain activity in CH36 and CH41 by tSMS may have caused the reduction in SFA and the improvement in DM perspective. However, as the rFPA is associated with various cognitive processes beyond SFA, even in social settings, unmeasured variables may also be affected by the suppression of the rFPA. For example, researchers have noted that prefrontal cortex activity in patients with SAD may reflect nonfunctional cognitive activity, such as inhibition (Brühl et al., 2014). Although the stimulation site was determined at the rFPA due to our purpose to investigate whether tSMS reduces SFA in social settings in this study, future research should determine whether other psychological indicators change when the tSMS is applied to the rFPA in similar settings.

Traditionally, cognitive-behavioral therapies have been widely used to modify SFA and mental perspective images in SAD. In the realm of NIBS, there have been two case reports (Paes et al., 2013) and a double-blind within-subject protocol (Heeren et al., 2017). The former study (Paes et al., 2013) using the low-rTMS over the right medial PFC combined with high-rTMS over the left MPFC for at least 4 weeks on consecutive weekdays found a decrease in Beck Depression Inventory (Beck et al., 1961), Beck Anxiety Inventory (Beck and Emery, 1985), and LSAS scores from baseline to follow-up. The latter study (Heeren et al., 2017) investigated the causal influence of left DLPFC neuromodulation on attention bias among 19 female individuals with a DSM-5 diagnosis of SAD. They adopted a double-blind within-subjects protocol in which they delivered a single session of anodal versus sham tDCS over the left DLPFC while completing a probe discrimination task assessing attention bias. As a result, participants demonstrated a significant decrease in attention bias during the anodal tDCS over the left DLPFC relative to the sham stimulation. These findings highlight tDCS as an innovative procedure to gain new insight into the underlying mechanisms of SAD. Overall, these studies have produced preliminary evidence for the effect of right prefrontal inhibition/left excitation on social anxiety symptoms. If tSMS, which is implemented more cost-effectively and with a lower burden than rTMS or tDCS, proves to be helpful, it can be one of the promising treatment strategies for SAD in the future.

It was also demonstrated that the intervention of Mindfulness-Based Stress Reduction with transcranial direct current stimulation for cognitive symptoms of depression or anxiety held potential effects on everyday mindfulness and social functioning (Brooks et al., 2021). Vedeniapin et al. (2010) also combined cognitive-behavioral therapy with rTMS. Besides, frontal hyperactivity compared to healthy controls has been reported in some other psychological disorders. For example, in depression, excessive activity in the right DLPFC has been reported (e.g., Grimm et al., 2008). Therefore, interventions using tSMS may apply to other psychological disorders as well.

This study has some limitations. The first limitation is the relatively small sample size. The lack of significant effects of tSMS on certain variables may be attributed to the small sample size. The second limitation is the absence of a direct clinical population. Although we demonstrated that the effects of tSMS become increasingly significant with the increase in social anxiety symptoms across several variables, this study did not directly target a clinical population. Therefore, it is still being determined whether tSMS would demonstrate greater efficacy in clinical populations or if its effects would be less pronounced. The third limitation is the unclear duration of the observed effects. If considering future clinical applications, it would be preferable for the effects to last longer. The fourth limitation is the need for more clarity regarding the remote brain effect. The researcher reported that tSMS can not only suppress brain function directly under the magnet but also induce various remote effects through brain networks (Shibata et al., 2021; Takamatsu et al., 2021). However, this study only measured the rPFA cortex, and the impact of tSMS on this area that extends to other brain regions warrants further investigation.

Our future research should address the following several key areas. First, we should continue to validate the effectiveness of tSMS for rFPA activity and for SFA using a larger sample. Second, we should investigate this for clinical populations. Using a more diverse sample would enable the generalization of the results and increase the feasibility of clinical applications. Third, we should investigate the duration of the effects. Investigating whether short-term changes in neural activity can lead to long-term neural plasticity is an important research question for clinical applications. Then, it is necessary to investigate the potential for long-lasting effects with multiple sessions of tSMS compared to a single session while also considering the safety of repeated interventions. Fourth, it would be helpful to investigate the various remote effects of tSMS on other brain regions and psychological indicators not measured in this study when tSMS is applied to the rFPA. Fifth, it should be considered whether different results would be obtained if the same experiment were conducted using tSMS with a single magnet system instead of the triple tSMS used in this study. These multifaceted approaches will significantly contribute to our understanding of tSMS’s therapeutic potential and its mechanism of action in psychological disorders.

## 5 Conclusion

This study represents an innovative use of tSMS to target SFA in social anxiety, demonstrating the potential effectiveness of tSMS intervention in improving the clinical symptoms of psychological disorders for the first time. The study’s comprehensive assessment approach, which combines functional near-infrared spectroscopy and questionnaires, provides a detailed understanding of the effects of the intervention on both brain activity and subjective experiences. tSMS demonstrated the suppression of activity in the rFPA, reducing associated SFA. Moreover, adaptive psychological functions such as the DM and field perspectives were enhanced. These results help close the gaps in our knowledge about the interaction between brain activity in the rFPA and SFA in social settings. They reveal a reciprocal relationship where rFPA activity can also influence SFA levels. Therefore, the findings of this study provide valuable insights for proposing tSMS as a novel approach for addressing social anxiety in the future. With potential clinical implications for treating social anxiety, this research contributes valuable insights to the neuroscience and mental health fields, offering a promising avenue for addressing the core symptoms of social anxiety disorders through novel brain stimulation techniques.

## Data availability statement

The datasets generated during and/or analyzed during the current study are available from the corresponding author on reasonable request.

## Ethics statement

The studies involving humans were approved by the Ethics Review Committee on Research with Human Subjects of Waseda University. The studies were conducted in accordance with the local legislation and institutional requirements. The participants provided their written informed consent to participate in this study.

## Author contributions

NT: Writing – original draft, Writing – review & editing, Conceptualization, Formal analysis, Funding acquisition, Methodology, Resources. HK: Conceptualization, Data curation, Investigation, Methodology, Writing – review & editing. YK: Conceptualization, Data curation, Investigation, Methodology, Writing – review & editing. TT: Conceptualization, Methodology, Writing – review & editing. SS: Conceptualization, Funding acquisition, Methodology, Resources, Writing – review & editing. TM: Conceptualization, Funding acquisition, Methodology, Resources, Writing – review & editing. RO: Conceptualization, Formal analysis, Funding acquisition, Methodology, Project administration, Resources, Supervision, Writing – review & editing. HK: Conceptualization, Formal analysis, Methodology, Project administration, Supervision, Writing – review & editing.
